# Genome-wide transcriptome and gene family analysis reveal candidate genes associated with potassium uptake of maize colonized by arbuscular mycorrhizal fungi

**DOI:** 10.1186/s12870-024-05398-6

**Published:** 2024-09-06

**Authors:** Yunjian Xu, Yixiu Yan, Tianyi Zhou, Jianhui Chun, Yuanchao Tu, Xinyu Yang, Jie Qin, Luyan Ou, Liang Ye, Fang Liu

**Affiliations:** 1 Ministry of Education Key Laboratory for Transboundary Ecosecurity of Southwest China, Yunnan Key Laboratory of Plant Reproductive Adaptation and Evolutionary Ecology and Centre for Invasion Biology, Institute of Biodiversity, School of Ecology and Environmental Science, Yunnan University, Kunming, 650504 Yunnan China; 2 School of Agriculture, Yunnan University, Kunming , Yunnan, 650504 China

**Keywords:** Mycorrhizae, Potassium deficiency, RNA-seq, Gene family

## Abstract

**Background:**

Potassium (K) is an essential nutrient for plant growth and development. Maize (*Zea mays*) is a widely planted crops in the world and requires a huge amount of K fertilizer. Arbuscular mycorrhizal fungi (AMF) are closely related to the K uptake of maize. Genetic improvement of maize K utilization efficiency will require elucidating the molecular mechanisms of maize K uptake through the mycorrhizal pathway. Here, we employed transcriptome and gene family analysis to elucidate the mechanism influencing the K uptake and utilization efficiency of mycorrhizal maize.

**Methods and results:**

The transcriptomes of maize were studied with and without AMF inoculation and under different K conditions. AM symbiosis increased the K concentration and dry weight of maize plants. RNA sequencing revealed that genes associated with the activity of the apoplast and nutrient reservoir were significantly enriched in mycorrhizal roots under low-K conditions but not under high-K conditions. Weighted gene correlation network analysis revealed that three modules were strongly correlated with K content. Twenty-one hub genes enriched in pathways associated with glycerophospholipid metabolism, glycerolipid metabolism, starch and sucrose metabolism, and anthocyanin biosynthesis were further identified. In general, these hub genes were upregulated in AMF-colonized roots under low-K conditions. Additionally, the members of 14 gene families associated with K obtain were identified (ARF: 38, ILK: 4, RBOH: 12, RUPO: 20, MAPKK: 89, CBL: 14, CIPK: 44, CPK: 40, PIN: 10, MYB: 174, NPF: 79, KT: 19, HAK/HKT/KUP: 38, and CPA: 8) from maize. The transcript levels of these genes showed that 92 genes (ARF:6, CBL:5, CIPK:13, CPK:2, HAK/HKT/KUP:7, PIN:2, MYB:26, NPF:16, RBOH:1, MAPKK:12 and RUPO:2) were upregulated with AM symbiosis under low-K conditions.

**Conclusions:**

This study indicated that AMF increase the resistance of maize to low-K stress by regulating K uptake at the gene transcription level. Our findings provide a genome-level resource for the functional assignment of genes regulated by K treatment and AM symbiosis in K uptake-related gene families in maize. This may contribute to elucidate the molecular mechanisms of maize response to low K stress with AMF inoculation, and provided a theoretical basis for AMF application in the crop field.

**Supplementary Information:**

The online version contains supplementary material available at 10.1186/s12870-024-05398-6.

## Introduction

Potassium (K) is an essential macronutrient for plants. It participates in many physiological processes, such as enzyme activation, photosynthesis, membrane transport, regulation of opening and closing of the stomata, and determining the yield and quality of crops [1, 2]. While the total K content in soils is quite large, the amounts available for plants are relatively small at any one time. This is because almost all K exists in the structural component of soil minerals and cannot be used for plants. The concentration of available potassium ions (K^+^) in soil is generally 0.025 mM to 5 mM [3]. Therefore, most plants suffer from low-K^+^ stress during their growth.

Potassium deficiency significantly affects plant growth and development [4]. To improve their K utilization efficiency (KUE), plants have evolved multiple complex uptake mechanisms [5]. The formation of symbiosis with arbuscular mycorrhizal fungi (AMF) is one of the most efficient methods [6]. AMFs are soil microorganisms that can form mutualistic symbioses with most terrestrial plants and improve their nutrient status [7]. A meta-analysis of the effects of AMF on nutrient uptake revealed that AMF could increase plant K uptake by 18.5% [8]. Kafle et al*.* have assessed the movement of K^+^ in AM symbiosis and suggested the existence of a mycorrhizal uptake pathway for K^+^ nutrition in *Medicago truncatula* [9]. When plants are colonized with AMF, they have access to a larger resource area. AMF develop a dense network of extraradical hyphae that extend the depletion zone around the roots and facilitate the acquisition of low-mobility nutrients, thus improving plant nutrient uptake [10, 11]. Moreover, AMF enhance nutrient uptake by regulating changes in host plants [8]. For example, AMF enhance nutrient uptake by regulating host root development. AMF colonization induces lateral root development in angiosperms via the expression of *CERK1*, *CEBiP*, and *NFR5* [12]. AM symbiosis improves tomato lateral root formation by modulating CEP2 peptide expression [13]. AMF also improve plant K uptake by upregulating K^+^ transporters. Using whole-genome RNA sequencing, a study revealed that several genes encoding putative transporters were upregulated in mycorrhizal *M. truncatula* plants under K^+^ deprivation [14]. Another study showed that a KT/KUP/HAK transporter, SlHAK10, which is upregulated in arbuscular mycorrhizal roots, improves K^+^ nutrition in tomato plants through the mycorrhizal pathway [15].

Transcriptome analysis has been used in many studies to explore mechanisms [16–19], including how plants respond to K^+^ deficiency. For example, transcriptomic analysis of Arabidopsis under low-K^+^ stress revealed many candidate genes related to low-K^+^ perception and regulatory pathways associated with responses to K^+^ deficiency [20, 21]. Rice root transcriptome analysis indicated that many genes, such as those in the protein kinase and ion transporter families, can play important roles during K^+^ deficiency [18]. Furthermore, a comparative analysis of transcriptomic changes between Arabidopsis and rice showed that monocots and dicots share many similar mechanisms in response to K^+^ deficiency [18]. In grapevines, the transcriptome reveals numerous differentially expressed genes (DEGs) and multiple response pathways to K^+^ deficiency treatment [19]. However, very little progress has been made in understanding changes in maize gene transcription under different K^+^ conditions with/without AMF colonization. The transcriptome provides promising tools for studying the contributions of mycorrhizae to K^+^ absorption in plants.

Maize (*Zea mays*) is a crop distributed worldwide for food, livestock feed, biofuel, and raw industrial material. Maize requires a large amount of K during its growth, but the efficiency of K uptake/utilization by maize cultivars is relatively low [22]. Arbuscular mycorrhizal symbiosis promotes the accumulation of K in maize [23]. However, the effects of AMF inoculation on K uptake under different K conditions and the transcriptional changes in maize genes under different K conditions in mycorrhizal roots are still unknown. Given the importance of AMF in the regulation of K uptake, the interactive impact of AMF on K uptake should be studied. Therefore, to better understand the regulatory effect of AMF on maize K uptake under low-K stress, candidate genes associated with AMF under different K conditions were screened via RNA sequencing analysis. In addition, important response pathways and gene families associated with K absorption were identified.

## Materials and methods

### Experimental materials and treatments

The plant material for this experiment was maize B73. The maize seeds were surface sterilized with 75% ethyl alcohol for 5 min and then washed with ddH_2_O 5 times. After washing, the seeds were germinated on moist filter paper at 28 °C for 3 days. After germination, plants at consistent growth stages were selected and transferred to plastic pots (28 × 25 cm) with two plants per pot. The planting medium was a 6:1:1 sterilized mixture of vermiculite, sand, and perlite.

The experiments included treatments in which AMF were inoculated or not inoculated at four concentrations of K (0.6 mM, 3 mM, 6 mM, or 9 mM KCl) for a total of eight treatments. AMF inoculation was achieved by adding *Rhizophagus irregularis* inoculum. The inoculum was provided by the National Engineering Lab of Crop Stress Resistance Breeding, Anhui Agricultural University, Anhui Province, China. A total of 1000 AMF spores per pot were added for the AMF inoculation treatment. The maize plants were grown in a greenhouse at 28 °C/25 °C under a 16-h day/8-h night cycle for five weeks. Plants received distilled water and a Hoagland nutrient solution free of K once per week. Five replications were established for each treatment.

### Plant analyses

Plant samples were harvested to measure dry weight, shoot length, K concentration, and the root colonization rate. The maize plants were dried at 105 °C for 30 min and then dried at 75 °C to a constant weight to measure the dry weight. The dried roots and shoots were ground and sieved (0.25 mm) to determine the K concentration. For the determination of the K concentration, ground samples were digested by adding H_2_SO_4_ with H_2_O_2_, and then the K concentration was determined using the flame photometer method.

Mycorrhizal colonization was analyzed after trypan blue staining according to previous reports [24]. In summary, the following steps were used for trypan blue staining: root excision into 1 cm long segments, fixation in FAA for 4 h, treatment with 10% KOH, heating at 90 °C for 1 h, treatment with a solution of 2% HCl for 5 min, staining with 0.05% trypan blue solution, and clearing with lactic acid and glycerin. After staining, the root fragments were mounted on slides, and the AMF structures were examined using a Leica DM5000B microscope. The root colonization rate was calculated using the root segment colonization weighting method [25].

### RNA sequencing

The roots of mycorrhizal and nonmycorrhizal maize plants were harvested five weeks after inoculation. Three biological replicates for each treatment were used for RNA sequencing, for a total of 24 samples. Total RNA was isolated using the RNAiso Plus Kit (TAKARA BIO INC.). The construction of the sequencing library and RNA sequencing were performed by Sangon (Shanghai, China) using an Illumina HiSeq 2500 platform. RNA-seq workflow consist of the following five steps: (1) preprocessing of raw data, (2) read alignment, (3) transcriptome reconstruction, (4) expression quantification, and (5) differential expression analysis. Firstly, RNA-seq data was subjected to quality control (QC) of the raw data before data analysis. Then, reads were assigned to the reference genome of maize B73 (Zm-B73-REFERENCE-NAM-5.0) using HISAT2 software after filtering out adaptor sequences and low-quality reads from the raw data. Next, transcriptome reconstruction was carried out by StringTie to identify all transcripts expressed in maize roots based on read mapping data. Last, differential expression analysis is conducted using DESeq2. Gene ontology (GO) functional annotation and Kyoto Encyclopedia of Genes and Genomes (KEGG) pathway enrichment analyses were performed using ClusterProfiler software.

### Quantitative real-time reverse transcription PCR (RT-qPCR)

RNA-Seq data was validated by RT-qPCR. DNase was used to eliminate potential trace contaminants of genomic DNA in RNA samples. The Hiscript II Reverse Transcriptase Kit (Vazyme, China) was used for cDNA synthesis, which was then used as templates for RT-qPCR. The RT-qPCR was performed on QuantStudio™ 7 Flex Real-Time PCR System. The Hieff UNICON® Universal Blue qPCR SYBR Green Master Mix kit was used (Yeasen Biotechnology, Shanghai, China) for qRT–PCR. Three biological replicates of each samples were performed. The primers used in this experiment are shown in Table S1. Reference genes *ZmACTIN1* and *ZmTUB* were used as internal control. Relative expression levels were calculated using the 2^–ΔΔCt^ method.

### Construction of module-trait relationships

Co-expression networks with highly connected expression patterns were constructed using the weighted gene co-expression network analysis (WGCNA) package in R according to previous studies [26, 27]. Gene expression data were standardized based on log2 (1 + FPKM) values and then imported into WGCNA to construct coexpression modules. Briefly, all gene expression data were normalized based on log2 (1 + FPKM) values, and then an adjacency matrix was generated. The adjacency matrix was converted to a topological overlap matrix (TOM), and genes were hierarchically clustered depending on the dissimilarity between the genes.

## Results

### Growth and K accumulation of AMF-inoculated maize under different K conditions

**Fig. 1:**
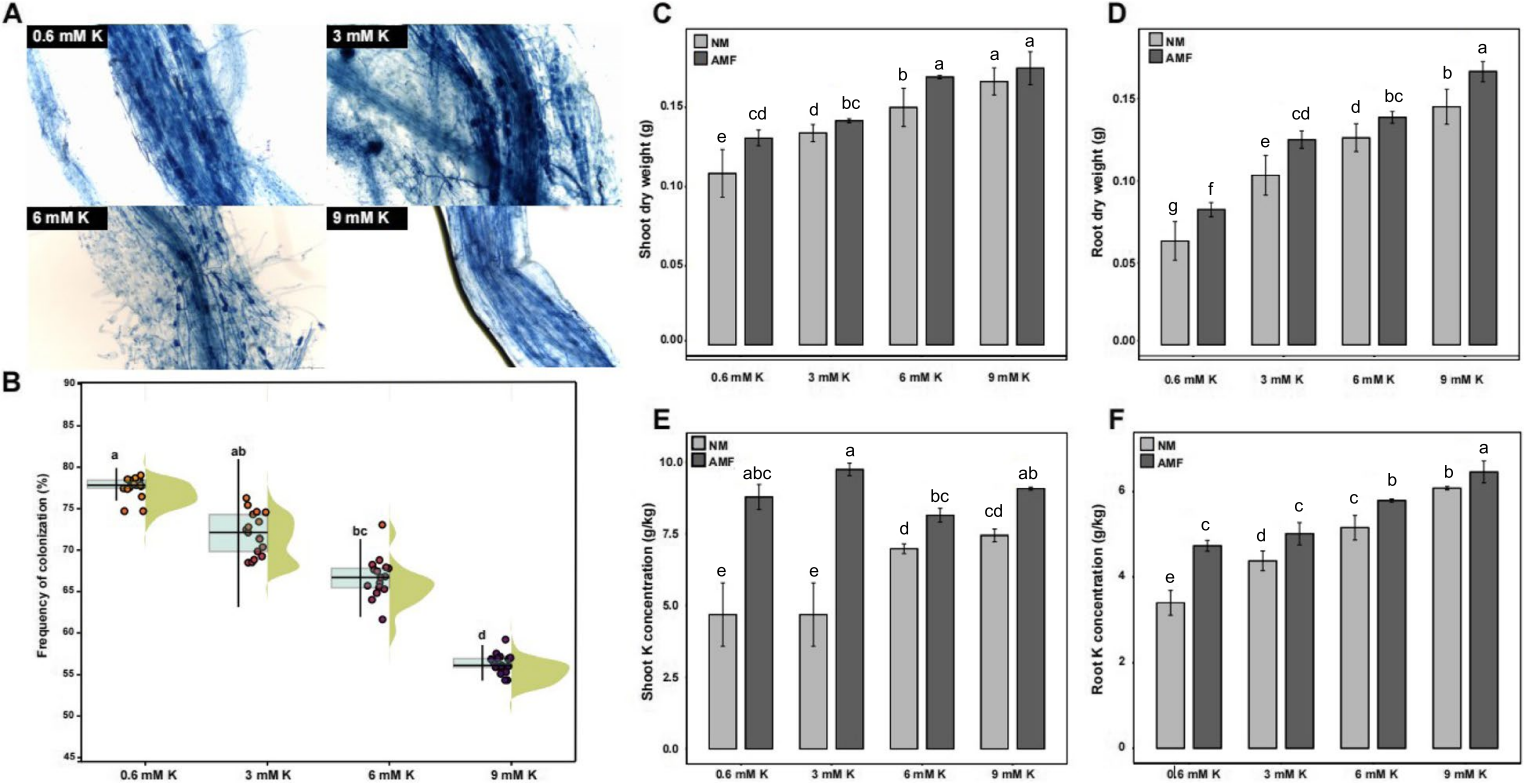
Effects of AMF inoculation on maize growth and K uptake under different K conditions. **A** Trypan blue staining of maize root. **B** Colonization rate of maize root under different K conditions. **C** Dry weight of shoot. **D** Dry weight of roots. **E** Concentrations of K in the shoot. **F** Concentrations of K in the roots. AMF, inoculated with arbuscular mycorrhizal fungi (*Rhizophagus intraradices*); NM, not inoculated with AMF. Different letters indicate significant differences between treatments according to two-way ANOVA followed by LSD post hoc tests (*P* < 0.05)

AMF successfully colonized maize roots and had different colonization rate with maize under different K conditions (Fig. 1A, B). Lower K conditions showed more AMF colonization, and AM symbiosis improved maize growth. Compared to noninoculated plants, maize plants inoculated with AMF exhibited obviously different phenotypes under low-K conditions (0.6 mM), especially in terms of leaf K deficiency symptoms (Fig. S1). The experiment showed that inoculation with AMF promoted maize plant growth (Fig. 1C, D). Under 0.6 mM (low) K, compared with no AMF inoculation, AMF inoculation significantly increased the shoot and root dry weights of maize by 27.74% and 16.61%, respectively. Under 3 mM (low) K conditions, the shoot and root dry weights significantly increased by 95.09% and 76.99%, respectively, after inoculation with AMF. Under 6 mM (normal) K conditions, the dry weights of the shoots and roots of maize increased by 50.09% and 100.33%, respectively, after AMF inoculation. However, under 9 mM K, no positive effect on the dry weight of maize plants was observed after AMF inoculation.

Additionally, compared with high-K conditions, lower-K conditions caused a reduction in the K content of the maize shoots and roots, but inoculation with AMF increased the K content of the maize plants (Fig. 1E, F). Under 0.6 mM (low) K conditions, compared with noninoculation, inoculation with AMF significantly increased the shoot and root K contents of maize by 38.15% and 55.65%, respectively. Under 3 mM (low) K conditions, AMF inoculation significantly increased the shoot and root K contents of maize by 120.30% and 59.04%, respectively. Under 6 mM (normal) K conditions, the K content of the shoot and root increased by 46.61% and 106.24%, respectively, after AMF inoculation. However, under 9 mM K, no positive effect on the K content of maize plants was observed after AMF inoculation (Fig. 1E, F). Therefore, inoculation with AMF could help maize cope with K deficiency in the soil and promote maize plant growth.

### Gene expression of mycorrhizal maize under different K conditions

**Fig. 2:**
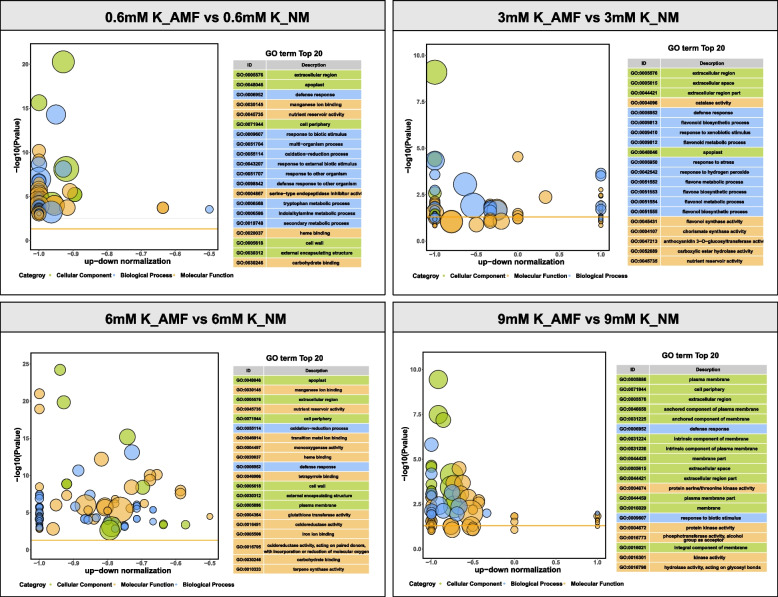
GO enrichment analysis of the differentially expressed genes in the four compared groups

To better understand the molecular, cellular, and biological effects of AMF inoculation on maize under different K conditions, we performed RNA sequencing (RNA-seq) analysis. A total of 1094, 1106, 1174, and 825 significantly upregulated genes (p value < 0.05) were identified after AMF inoculation under K concentrations of 0.6 mM, 3 mM, 6 mM, and 9 mM, respectively (Table S2). All upregulated genes were assigned to Gene Ontology (GO) and were classified by biological process (BP), molecular function (MF), and cellular localization (CC) (Fig. 2, Figs. S2-S4). Among the top 20 GO-enriched terms, the most commonly occurring annotations for AMF colonization in the four pairwise comparisons (0.6 mM K_AMF vs 0.6 mM K_NM, 3 mM K_AMF vs 3 mM K_NM, 6 mM K_AMF vs 6 mM K_NM, and 9 mM K_AMF vs 9 mM K_NM) were related to the defense response (BP) and extracellular region (CP). Further analysis revealed that apoplastic and nutrient reservoir activities were significantly enriched in the 0.6 mM K_AMF vs. 0.6 mM K_NM, 3 mM K_AMF vs. 3 mM K_NM, and 6 mM K_AMF vs. 6 mM K_NM treatments but not in the 9 mM K_AMF vs. 9 mM K_NM treatment.

To better understand the physiological processes of roots colonized by AMF under different K conditions, we mapped the DEGs to Kyoto Encyclopedia of Genes and Genomes (KEGG) pathways. KEGG enrichment analysis revealed that most of these genes whose expression changed were related to metabolic pathways and the biosynthesis of secondary metabolites pathways in the four pairwise comparisons (Fig. S5). In the 0.6 mM K_AMF treatment group versus the 0.6 mM K_NM treatment group, 76 and 67 genes whose expression changed were related to metabolic pathways and the biosynthesis of secondary metabolites, respectively. In 3 mM K_AMF versus 3 mM K_NM, 9 and 8 genes whose expression changed, respectively, were related to metabolic pathways and the biosynthesis of secondary metabolites. In the comparison of 6 mM K_AMF and 6 mM K_NM, 78 and 57 genes whose expression changed, respectively, were related to metabolic pathways and the biosynthesis of secondary metabolites. In the 9 mM K_AMF vs. 9 mM K_NM treatment, there were 11 and 6 genes related to metabolic pathways and the biosynthesis of secondary metabolites, respectively. Among the top 25 enriched pathways, the four comparison groups were enriched in the biosynthesis of secondary metabolites, flavonoid biosynthesis, and metabolic pathways (Fig. S5), while six pathways (including betalain biosynthesis, isoquinoline alkaloid biosynthesis, phenylalanine metabolism, phenylalanine, tyrosine and tryptophan biosynthesis, tryptophan metabolism, and tyrosine metabolism) were not enriched in the 9 mM K_AMF group compared with the 9 mM K_NM group.

### Gene co-expression analysis during AM symbiosis under different K conditions by weighted gene co-expression network analysis (WGCNA)

**Fig. 3:**
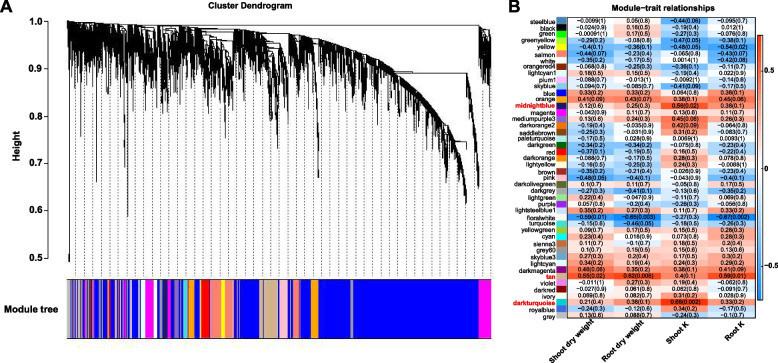
Weighted gene co-expression network analysis (WGCNA). **A** Gene cluster dendrogram based on the topological overlap of 46 different modules. Each module is marked with a different color, and genes that did not belong to any module were put into the grey module. **B** Correlation analysis of the modules and traits. P values are shown in parentheses

To identify genes related to K uptake through the AM symbiosis pathway under different K conditions, we performed a weighted gene co-expression network analysis (WGCNA) using differentially expressed genes (DEGs). The genes were grouped into 46 co-expression modules (Fig. 3A). The expression patterns of all genes were distinguished and categorized to generate a cluster dendrogram with 46 modules (Fig. 3A). The three module-trait relationships significantly correlated with plant dry weight and K content in the 24 samples (Fig. 3B). The modules 'midnight blue' (*r* = 0.56, *p* = 0.02) and 'dark turquoise' (*r* = 0.68, *p* = 0.002) were positively correlated with the shoot K content. The “tan” module had a significant positive correlation with shoot dry weight (*r* = 0.55, *p* = 0.02), root dry weight (*r* = 0.62, *p* = 0.006) and root K content (*r* = 0.59, *p* = 0.01). On the basis of the above results, the three modules related to K uptake through the mycorrhizal pathway under different K conditions were selected for further analysis.

### Characteristics and hub genes of the transcriptional regulatory modules correlated with K uptake from the mycorrhizal pathway

**Fig. 4:**
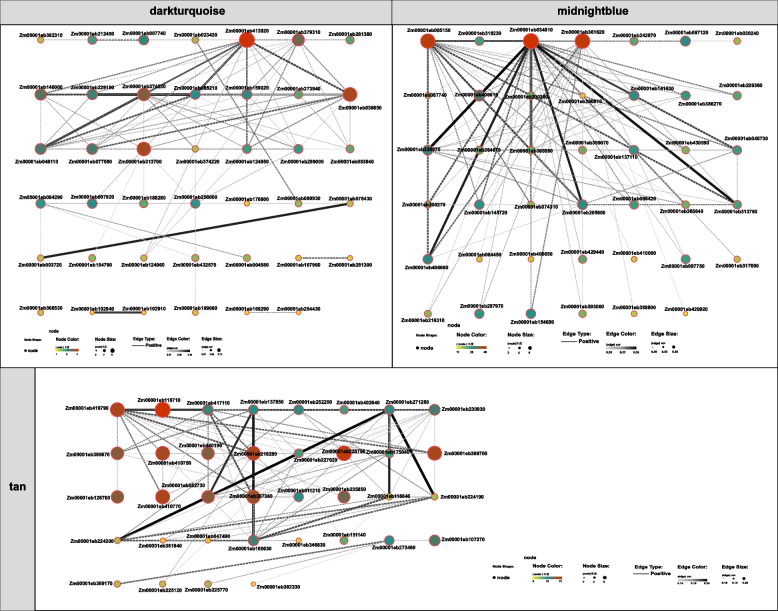
Transcriptional regulatory network analyses of genes in the midnightblue, darkturquoise and tan models. Circles represent the candidate genes (nodes). The color and size of each node represent the expression level of each gene. The solid and dotted lines (edges) represent positive and negative relationships between genes, respectively. The line color and size represent the weight

**Fig. 5:**
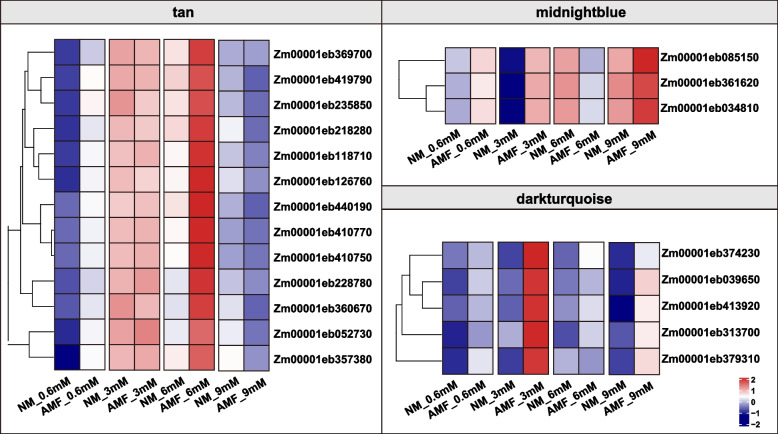
Heatmap showing the expression patterns of the candidate genes in the midnightblue, darkturquoise and tan models under different potassium conditions with and without AMF colonization. AMF represents arbuscular mycorrhizal fungal colonization; NM represents no AMF colonization

The midnight blue module was highly overrepresented, with genes annotated as related to the carotene biosynthetic process (GO: 0016120), sulfolipid metabolic process (GO: 0046505) and sulfolipid biosynthetic process (GO: 0046506) (Fig. S6). Among these genes, three genes in the midnightblue module were identified as hub genes, namely, *Zm00001eb085150*, *Zm00001eb034810* and *Zm00001eb361620* (Fig. 4). These genes had higher expression levels and a greater number of edges connecting to other genes. KEGG analysis consistently indicated that these genes are closely related to glycerophospholipid metabolism and glycerolipid metabolism. *Zm00001eb085150* and *Zm00001eb361620* are directly related to glycerophospholipid metabolism, and *Zm00001eb034810* is related to glycerolipid metabolism. The expression of *Zm00001eb085150*, *Zm00001eb034810* and *Zm00001eb361620* was significantly upregulated in roots inoculated with AMF under low-K conditions (0.6 mM and 3 mM) but was reduced in mycorrhizal roots under 6 mM K conditions (Fig. 5).

The tan module was enriched in proteins related to phenylpropanoid catabolic processes (GO: 0046271) and lignin catabolic processes (GO: 0046274) (Fig. S6). The expression levels of 13 hub genes, *Zm00001eb419790*, *Zm00001eb118710*, *Zm00001eb360670*, *Zm00001eb410750*, *Zm00001eb440190*, *Zm00001eb218280*, *Zm00001eb228780*, *Zm00001eb369700*, *Zm00001eb126760*, *Zm00001eb410770*, *Zm00001eb052730*, *Zm00001eb357380*, and *Zm00001eb235850*, drastically increased in mycorrhizal roots under 0.6 mM and 6 mM K but decreased in mycorrhizal roots under 9 mM K (Fig. 5). Among these genes, *Zm00001eb419790* was annotated as *ascorbate peroxidase 6* (*APX6*). *Zm00001eb228780* was annotated as *sucrose synthase 7* (*SUS7*), and *Zm00001eb360670* was annotated as *reticulon-like1*.

Furthermore, proteins involved in the flavone biosynthetic process (GO: 0051553, 0051555) and flavone metabolism (GO: 0051552, 0051554) were overrepresented in the dark turquoise module (Fig. S6). Five hub genes were identified in this module, namely, *Zm00001eb413920*, *Zm00001eb039650*, *Zm00001eb313700*, *Zm00001eb379310*, and *Zm00001eb374230* (Fig. 4). The expression levels of these genes in maize roots were almost upregulated after inoculation with AMF under all K conditions (0.6 mM, 3 mM, 6 mM and 9 mM) (Fig. 5). Among these genes, *Zm00001eb374230* was annotated as *bronze1* (*BZ1*), *Zm00001eb039650* was annotated as *cysteine protease35* (*CCP35*), and *Zm00001eb413920* was annotated as WRKY33. Among these hub genes from midnightblue module, tan module and dark turquoise module, RT-qPCR of eight genes was conducted to validate the credibility of the transcriptome data and to analyze the the expression patterns of the genes under K conditions of 0.6 mM, 3 mM, and 6 mM with/without AMF inoculation. The results showed that the expressional trends of these genes were highly consistent with the transcriptome results (Fig. S7).

### Identification of gene families involved in low K signal transduction and uptake associated with AM symbiosis

**Fig. 6:**
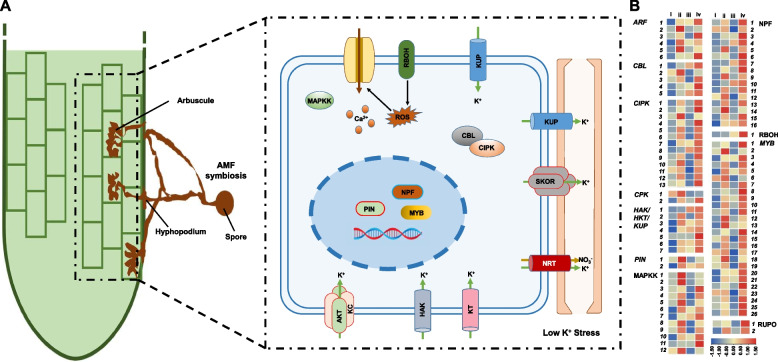
Response of maize root for AMF colonization under low K stress. **A** Schematic representation of genes in maize root response for AMF colonization under low K conditions. **B** Expression profiles of genes from eleven gene families associated with AMF colonization under low K conditions. The i, ii, iii, and iv represent the treatment of 0.6 mM K condition without AMF inoculation, 0.6 mM K condition with AMF inoculation, 3 mM K condition without AMF inoculation, and 3 mM K condition with AMF inoculation, respectively. Gene ID of each gene were list in Table S3

Many genes involved in signal transduction and the regulation of ion transporters in plant responses to low-K stress, such as genes in ARF family, CBL family, CIPK family, CPK family, ILK family, RBOH family, MAPKK family, RUPO family, NPF family, MYB family, PIN family, CPA family, KT family, and HAK/HKT/KUP family, have been reported [28]. We conducted a genome-wide identification of these gene families in maize by BLAST searches. In total, 38 members of ARF, 4 members of ILK, 12 members of RBOH, 20 members of RUPO, 89 members of MAPKK, 14 members of CBL, 44 members of CIPK, 40 members of CPK, 10 members of PIN, 174 members of MYB, 79 members of NPF, 19 members of KT, 38 members of HAK, and 8 members of CPA were identified (Table S3). We speculated that there is a similar pathway of maize root response for low K conditions with AMF colonization (Fig. 6A). Thus, we further explored these families genes response to AMF inoculation under low K conditions based on the RNA-seq data. As expected, we found many genes in these families respond to AMF colonization under low K stress. In total, 6 ARF genes, 5 CBL genes, 13 CIPK genes, 2 CPK genes, 7 HAK/HKT/KUP genes, 2 PIN genes, 26 MYB genes, 16 NPF genes, 1 RBOH gene, 12 MAPKK genes and 2 RUPO genes exhibited greater expression in roots with AMF colonization than in those without AMF colonization under low-K conditions (0.6 mM and 3 mM) (Fig. 6B). Among these genes, four HAK/HKT/KUP genes were selected to validate the transcriptome data by RT-qPCR, which results showed that the expression of these genes were largely consistent with the transcriptome results (Fig. S6). Further we performed phylogenetic analysis of these families gene with those reported gene with different functions (Figs. S8-S18). As we can see, many of the predictive genes in this study that may be associated with AM-associated K acquisition are not clustered with genes that have been reported to have other functions.

## Discussions

Symbiosis with AMF significantly increased maize plant growth and K concentrations under low-K conditions (Fig. 1B-E). However, under 9 mM K condition, there was no positive effect on the K content or growth of the maize plants after inoculation with AMF (Fig. 1B-E). These results indicated that inoculation with AMF can indeed help maize cope with K deficiency in the soil. The response to K deficiency has been well studied in many plants but has been less studied in plants with AM symbiosis. To this end, we investigated the molecular response mechanisms of maize to different K conditions with and without AMF inoculation via transcriptome analysis. By comparing the AMF-colonized (AM) and noncolonized (NM) samples under different K conditions, we found that AMF inoculation commonly affected the defense response under both low- and high-K conditions. AMF colonization-triggered basal defense responses have been reported in many studies [29], which reinforces the credibility of our data.

By comparing samples from relatively low-K conditions (0.6 mM, 3 mM, and 6 mM, relative to 9 mM) colonized or not colonized by AMF, we found that genes involved in nutrient reservoir activity were enriched in mycorrhizal roots, possibly indicating that AM symbiosis reallocated nutrient resources to plant growth under low-K conditions. In addition to the activity of the nutrient reservoir, the GO terms suggested that AMF regulate the cellular component in the apoplast in response to relatively low-K conditions. The apoplast plays an important role in diverse biological processes, including intercellular signaling, plant‒microbe interactions, and both water and nutrient transport [30]. Changes in the apoplast caused by AMF colonization may change the transmission of low-K signals, thus regulating K transport. Additionally, AMF affect the soil immediately surrounding their hyphae and the hyphosphere [31, 32] and recruit specific microbiota to the rhizosphere [33, 34], helping AMF acquire organic nutrients [31, 35, 36]. Since the apoplast can provide habitat for microorganisms [30], AMF hyphae might attract and transport some microorganisms to the plant host apoplast, which could contribute to K nutrition. Therefore, AMF can improve maize K status by promoting nutrient reservoir activity and apoplastic biosynthesis.

KEGG pathway analysis can help us to understand the physiological processes of roots colonized by AMF under different K conditions. Many studies have revealed pathways enriched in plants colonized with AMF via transcriptomic, proteomic, or metabonomic analysis. According to transcriptomic and proteomic analyses of soybean plants inoculated with AMF, key pathways and differentially expressed proteins have been found to be involved in processes associated with “metabolic pathways” [37]. Functional enrichment analyses of differentially accumulated metabolites and differentially expressed genes revealed the most differentially regulated pathways, including amino acid and sugar metabolism, secondary metabolite biosynthesis, and phenylpropanoid biosynthesis pathways [38]. Our study was consistent with these results; AMF inoculation also enriched the biosynthesis of secondary metabolites, flavonoid biosynthesis, and metabolic pathways, which were irrelevant to K conditions.

Moreover, we found different pathways enriched in mycorrhizal roots associated with low-K conditions, including the biosynthesis and metabolism of aromatic amino acids (tryptophan, tyrosine and phenylalanine), betalain biosynthesis and isoquinoline alkaloid biosynthesis (Fig. S5). In general, aromatic amino acids and related specialized metabolites increase due to abiotic stress and can contribute to tolerance to abiotic stress in plants [39–41]. In NM roots, genes involved in the biosynthesis and metabolism of aromatic amino acid pathways were upregulated, suggesting that Trp, Tyr, and Phe contribute to coping with low-K stress by promoting the biosynthesis of secondary metabolites. However, genes in these pathways were downregulated in AM-associated roots. AMF improved K levels and promoted maize growth, suggesting that AM symbiosis reduced low-K stress, resulting in reduced expression of genes associated with the biosynthesis and metabolism of aromatic amino acids. Betalains are synthesized from L-tyrosine [42], exhibit antioxidant activity through free radical scavenging and accumulate in response to different abiotic and biotic stresses [43–46]. Similar to the biosynthesis and metabolism of aromatic amino acids, AM symbiosis reduced the expression of genes associated with betalain biosynthesis but promoted the ability of maize to cope with low-K stress. Therefore, we speculated that colonization with AMF improved maize K status by promoting nutrient reservoir activity and apoplast biosynthesis, resulting in a reduction in stress caused by K deficiency.

By WGCNA, we identified three specific modules that may be related to K uptake through the AM pathway (Fig. 3B). GO analysis revealed that genes in the three modules were enriched for the carotene biosynthetic process, sulfolipid metabolic and biosynthetic process, phenylpropanoid catabolic process, lignin catabolic process, and flavone biosynthetic and metabolic processes (Fig. S6). Isoflavones are critical for successful symbiotic interactions between soybeans and rhizobia [47]. Phenylpropanoid compounds, flavonoids, monolignans, phenolic acids, stilbenes, and coumarins play crucial roles in plant development and enhancing resistance [48]. These compounds contribute to the plant's defense mechanisms against various stresses. The regulation of these key metabolic processes by AMF contributes to the increased resistance of maize to low-K stress.

Among the three specific modules, 21 hub genes were identified. Among them, three genes (*Zm00001eb085150*, *Zm00001eb361620* and *Zm00001eb085150*) were related to glycerophospholipid metabolism and glycerolipid metabolism. Glycerophospholipid metabolism is one of the key metabolic processes in soybean root systems affected by symbiosis [49]. Glycerophospholipid metabolism promotes early symbiosis between rhizobia and soybeans through extracellular vesicles, facilitating a symbiotic relationship [50]. In AM symbiosis, enhanced glycerophospholipid metabolism could ensure rapid colonization of maize roots by AMF under low-K stress, thus supporting normal maize growth. *Zm00001eb419790,* annotated as *ascorbate peroxidase 6* (*APX6*), was another hub gene. APX6 reportedly modulates ROS signaling via cross talk with hormone signals [50] and plays an important role in developmental and stress-induced senescence programs [51], suggesting that *Zm00001eb419790* may play a role in reducing ROS and promoting resistance to K deficiency in mycorrhizal roots.

Another hub gene, *Zm00001eb228780*, was annotated as *sucrose synthase 7* (*SUS7*), which may play a role in sucrose synthesis and degradation. AMF can affect sucrose-related enzymes to regulate sucrose synthesis and degradation in host plants for AM development, thus stimulating plant growth [52]. Furthermore, a previous study revealed that changes in sucrose metabolism caused by AM symbiosis via the regulation of sucrose-metabolized enzyme activities are one way for the host plant to undergo osmotic adjustment [53]. K^+^ affects the osmotic pressure in the root xylem, which drives long-distance sap flow from roots to shoots [54]. Thus, under low-K conditions, AM symbiosis could improve the K state of plants by regulating osmotic pressure through changes in sucrose metabolism. *Zm00001eb360670* was annotated as *reticulon-like1*, which is also a hub gene. Its homologous gene, Arabidopsis *reticulon-like* (*RTNLB*)-encoded protein, is located in the endoplasmic reticulum and is involved in endomembrane trafficking in plant cells [55]. Maize reticulon proteins 1 and 2 have been revealed to be receptors for autophagy-mediated ER turnover and are critical for ER homeostasis and the suppression of ER stress [56]. The Zm00001eb360670 protein may be involved in endomembrane K transport.

For other hub genes, for example, *Zm00001eb374230* was annotated as *bronze1* (*BZ1*). *BZ1* is the gene that encodes UFGT (flavonoid-3-O-glucosyl-transferase) [57], and studies have shown that anthocyanin accumulation is suppressed in maize *bz1 mutants* [58]. In grapes, during fruit development, potassium application promoted the accumulation of anthocyanins in fruit by regulating the up-regulation of genes expression, such as UFGT [59]. Therefore, in this study, the upregulation of *Zm00001eb374230* suggested that it may be involved in the enhancement of K obtained after AM symbiosis. *Zm00001eb413920* was annotated as WRKY33. WRKY members have been reported to play significant roles in abiotic stress response and secondary metabolite biosynthesis regulation [60, 61]. Under low-K conditions, AM symbiosis may promote maize resistance to low-K stress by upregulating the expression of the WRKY gene *Zm00001eb413920*. As a transcription factor, *Zm00001eb413920* can respond to low-K stress through any pathway.

To further elucidate the mechanism of K acquisition by maize under the low K condition with AM symbiosis, we further identified genes related to K signaling and transport pathways based on the previous study [28], and explored their response to AMF colonization under different K conditions. We found that except the ILK and KT genes, genes responsible for low K condition also showed responsible for AM colonization (0.6 mM and 3 mM K conditions), including genes from ARF, CBL, CIPK, CPK, RBOH, MAPKK, RUPO, NPF, MYB, PIN, CPA, and HAK/HKT/KUP. Phylogenetic analysis suggests that many of the predicted family genes may be related to the acquisition of K by AM symbiosis rather than having similar functions to other genes that have already been reported. Both the hub genes reported in this study and the genes involved in the K signal transduction pathway are likely the core regulatory genes for K deficiency responses in mycorrhizal maize, and an in-depth study of these genes will aid in understanding the molecular network involved in plant responses to K starvation.

## Conclusions

In conclusion, this study analyzed the transcriptome of maize roots with/without arbuscular mycorrhizal fungus (AMF) inoculation under different potassium (K) concentrations. Genes associated with the activity of the apoplast and the nutrient reservoir were enriched in mycorrhizal roots under low-K conditions. Many key genes were revealed to respond to low-K conditions with AM symbiosis. These genes were related to glycerophospholipid metabolism, glycerolipid metabolism, starch and sucrose metabolism, and anthocyanin biosynthesis. Additionally, gene families involved in K signal transduction and K uptake were further identified, and these genes were upregulated in mycorrhizal roots under low-K conditions. Thus, AMF increase the resistance of maize to low-K stress by improving the absorption of K by plants and regulating K uptake at the gene transcription level. Although the functions of these genes have been inferred from their homologous genes in many model plants, their multiple functions in maize associated with AM symbiosis still need to be investigated for genetic improvement of maize with efficient potassium utilization.

## Supplementary Information

Supplementary Material 1.

Supplementary Material 2.

Supplementary Material 3.

Supplementary Material 4.

## Data Availability

The datasets generated and analyzed during the current study are available in the NCBI SRA database (PRJNA1048535). All data generated or analyzed during this study are included in this published article and its supplementary information files.
