# Supplementary material for: Genome-wide transcriptome and gene family analysis reveal candidate genes associated with potassium uptake of maize colonized by arbuscular mycorrhizal fungi
Source: BMC Plant Biol. 2024 Sep 6;24:838. doi: 10.1186/s12870-024-05398-6 (PMC11378567; doi:10.1186/s12870-024-05398-6)
Supplement: Supplementary file 1 — Supplementary Material 1. [file 12870_2024_5398_MOESM1_ESM.pdf]

Supplementary figures

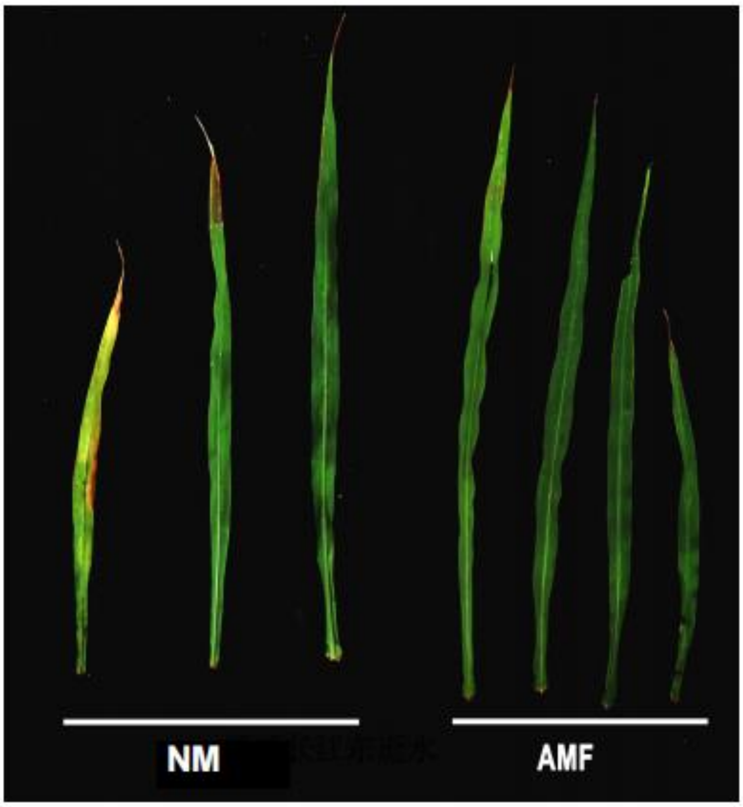

Fig. S1 Leaf phenotypes of maize with and without AMF inoculation under different K conditions.

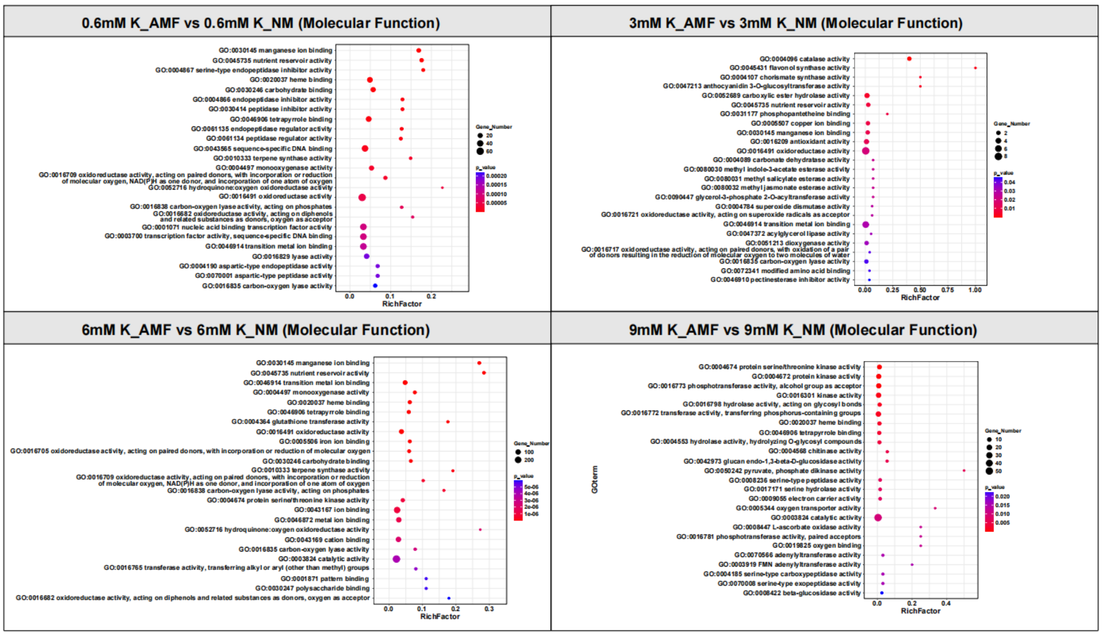

Fig. S2 Molecular function of GO enrichment.

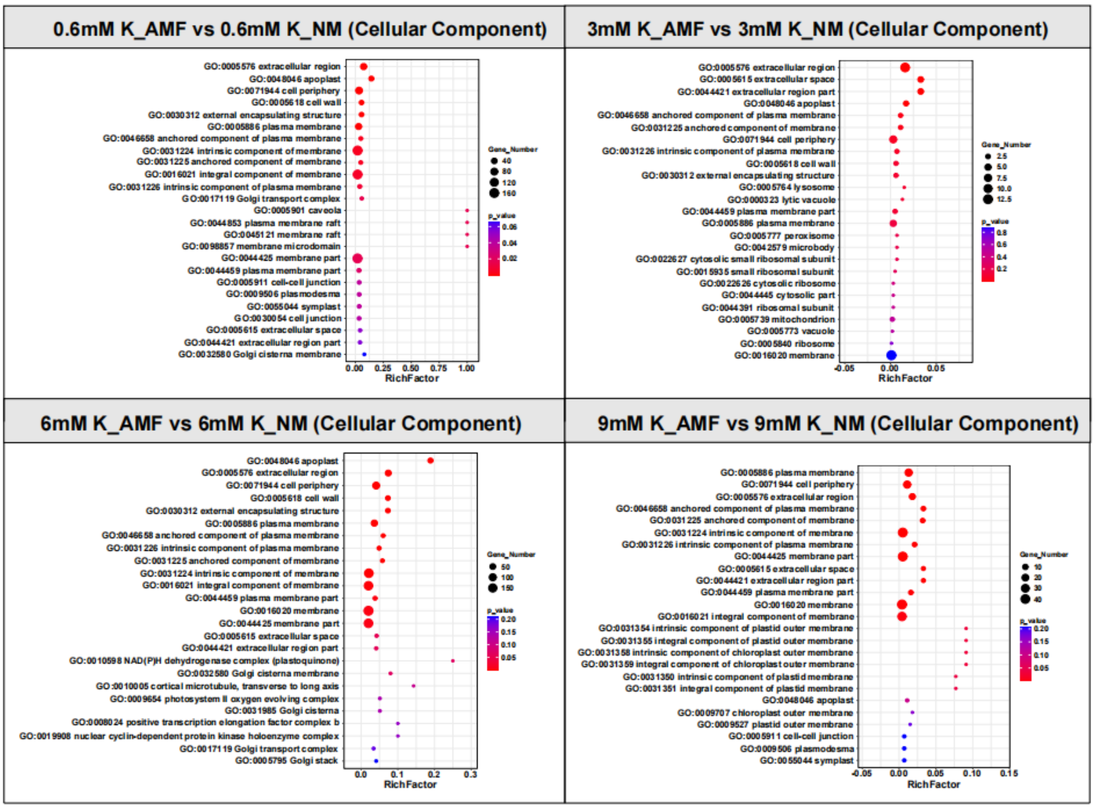

Fig. S3 Cellular components of GO enrichment.

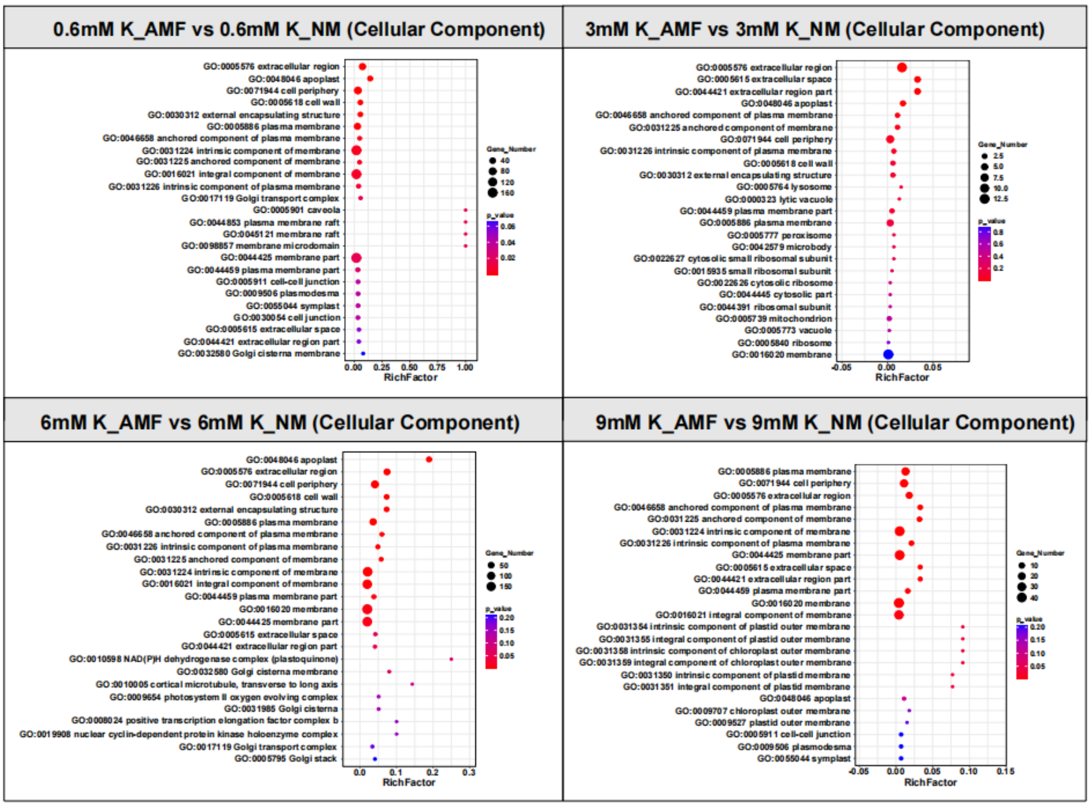

Fig. S4 Biological process of GO enrichment.

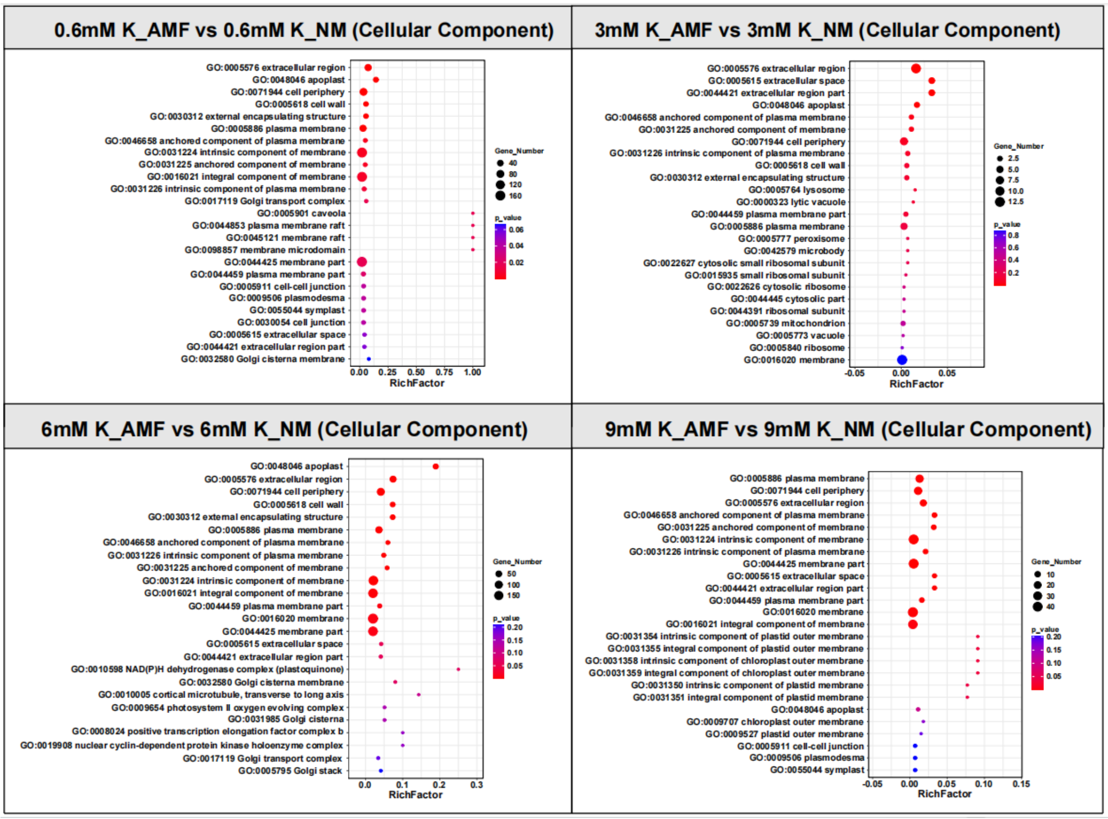

Fig. S5 The top 25 enriched KEGG pathways of the significantly changed genes.

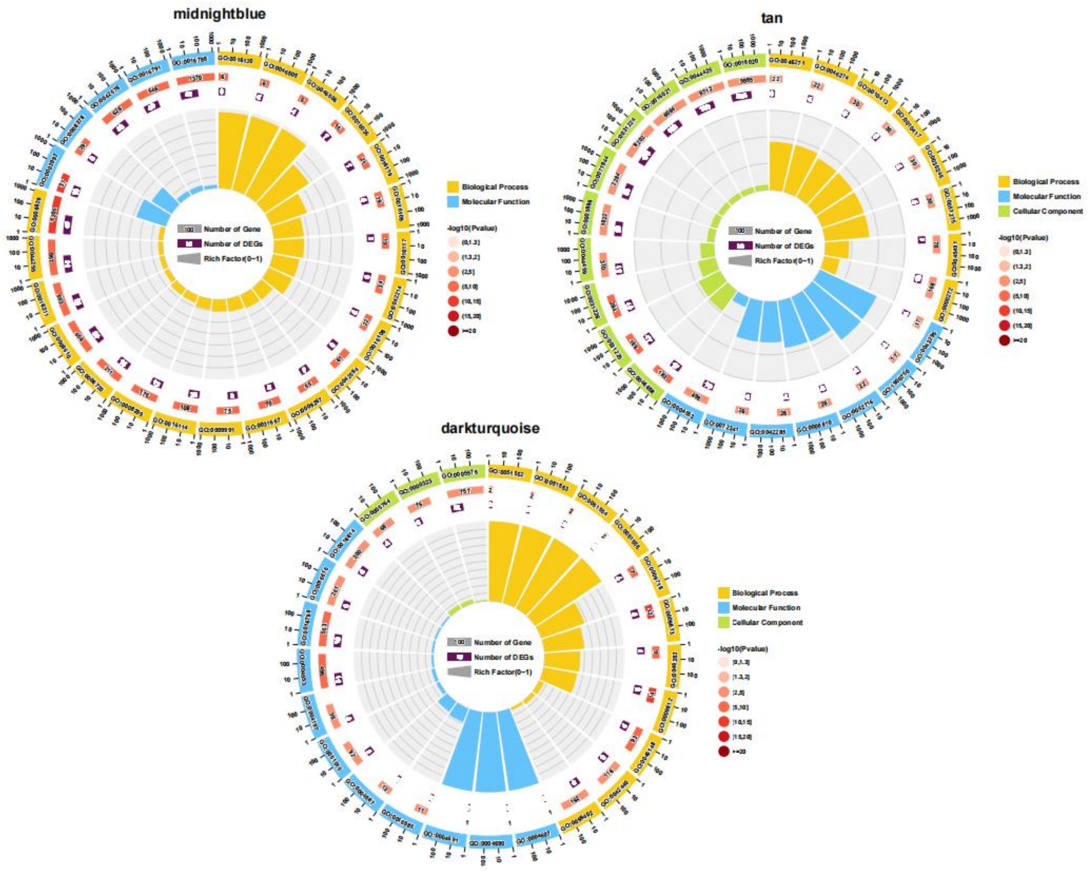

Fig. S6 GO analysis of genes in the midnightblue, darkturquoise and tan models.

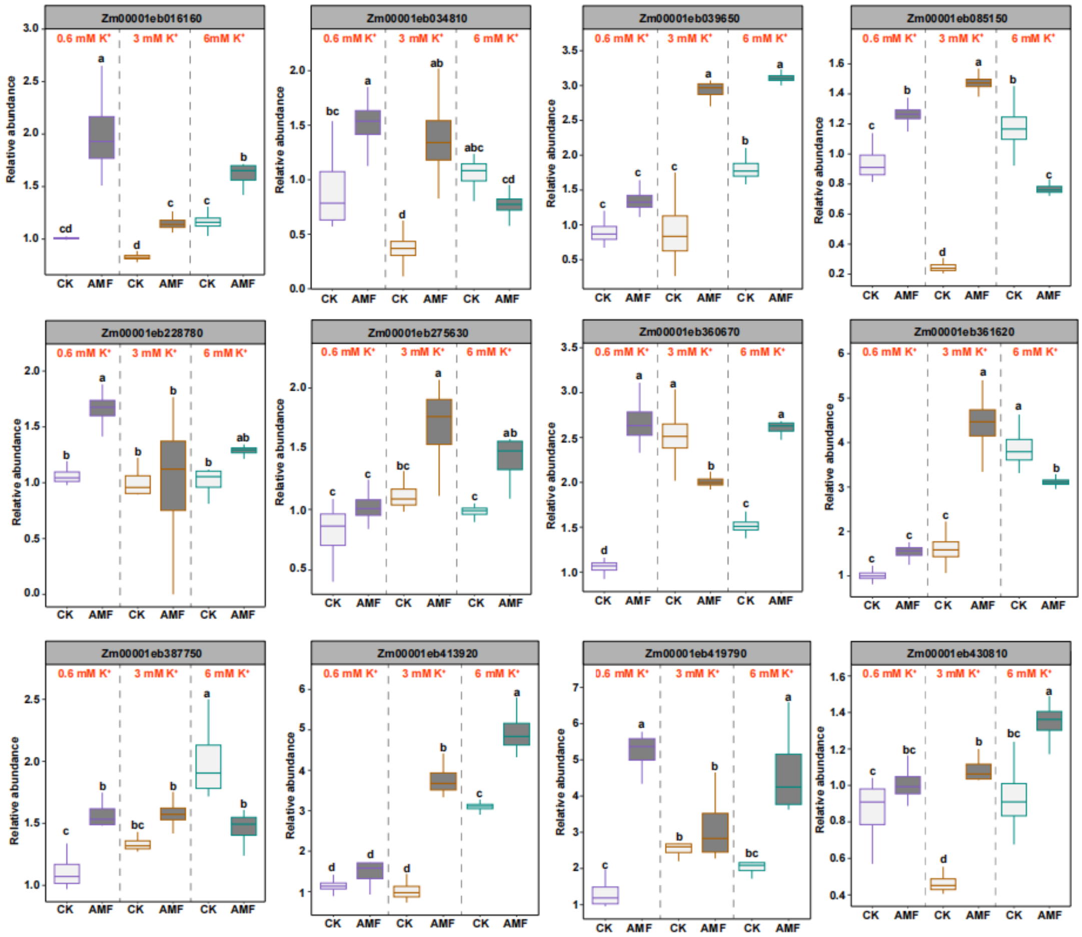

Fig. S7 The qRT-PCR results of 12 genes under K conditions of 0.6 mM, 3 mM, and 6 mM with/without AMF inoculation. The same letter indicates not statistically significant ($p>0.05$).

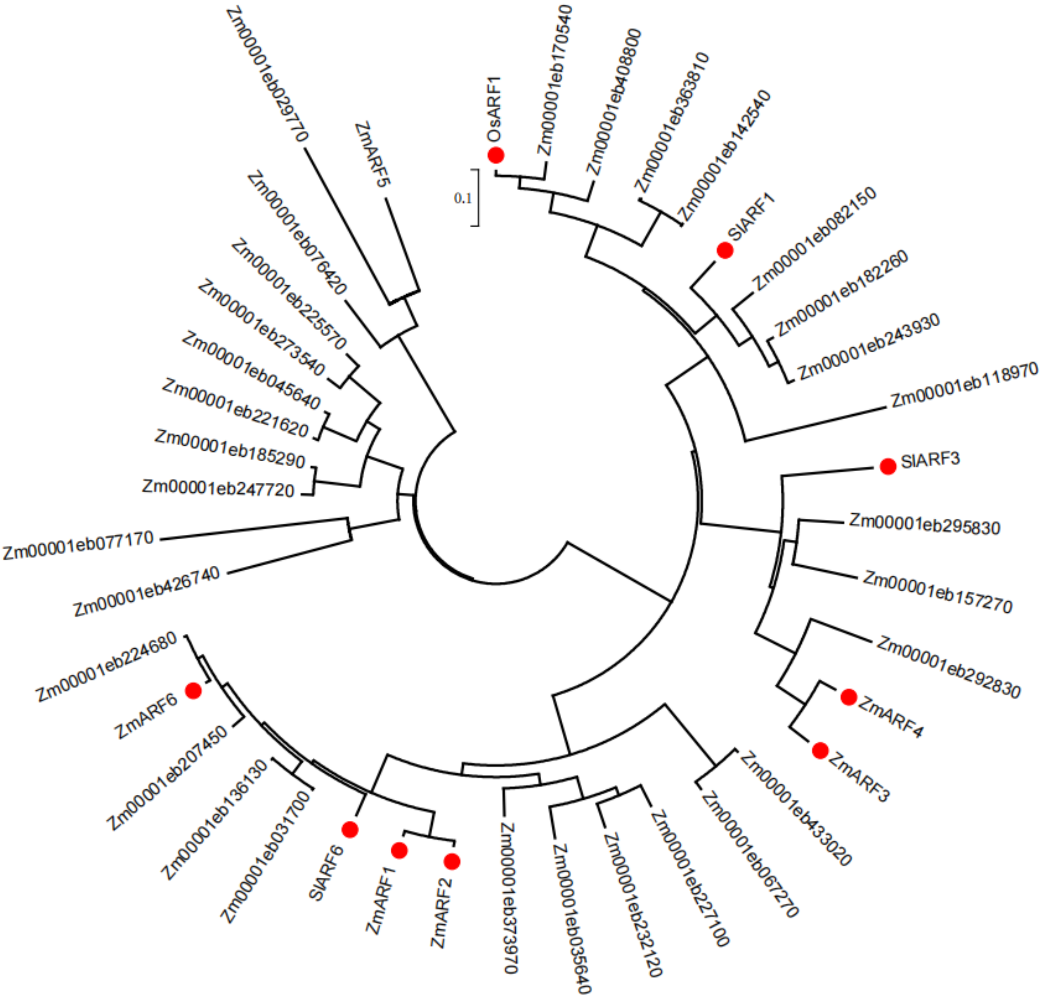

Fig. S8 Phylogenetic tree analysis of maize ARF family genes and reported genes.

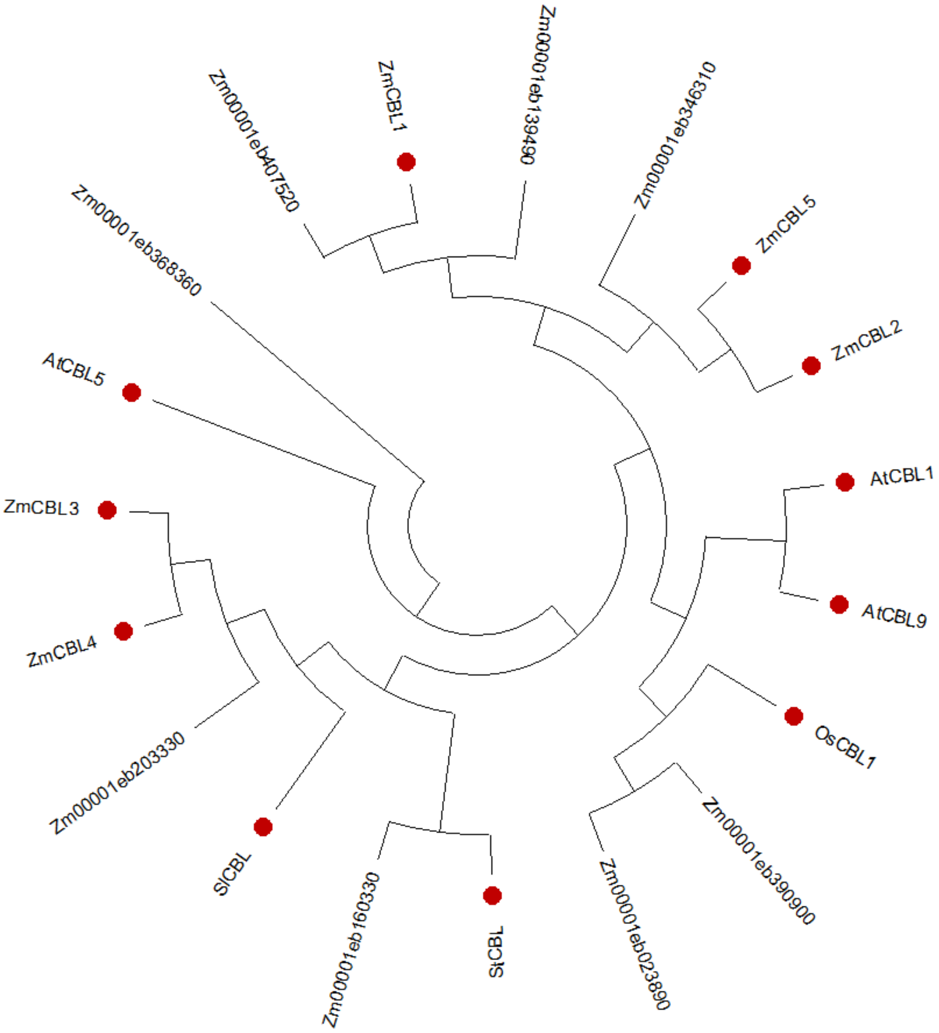

Fig. S9 Phylogenetic tree analysis of maize CBL family genes and reported genes.

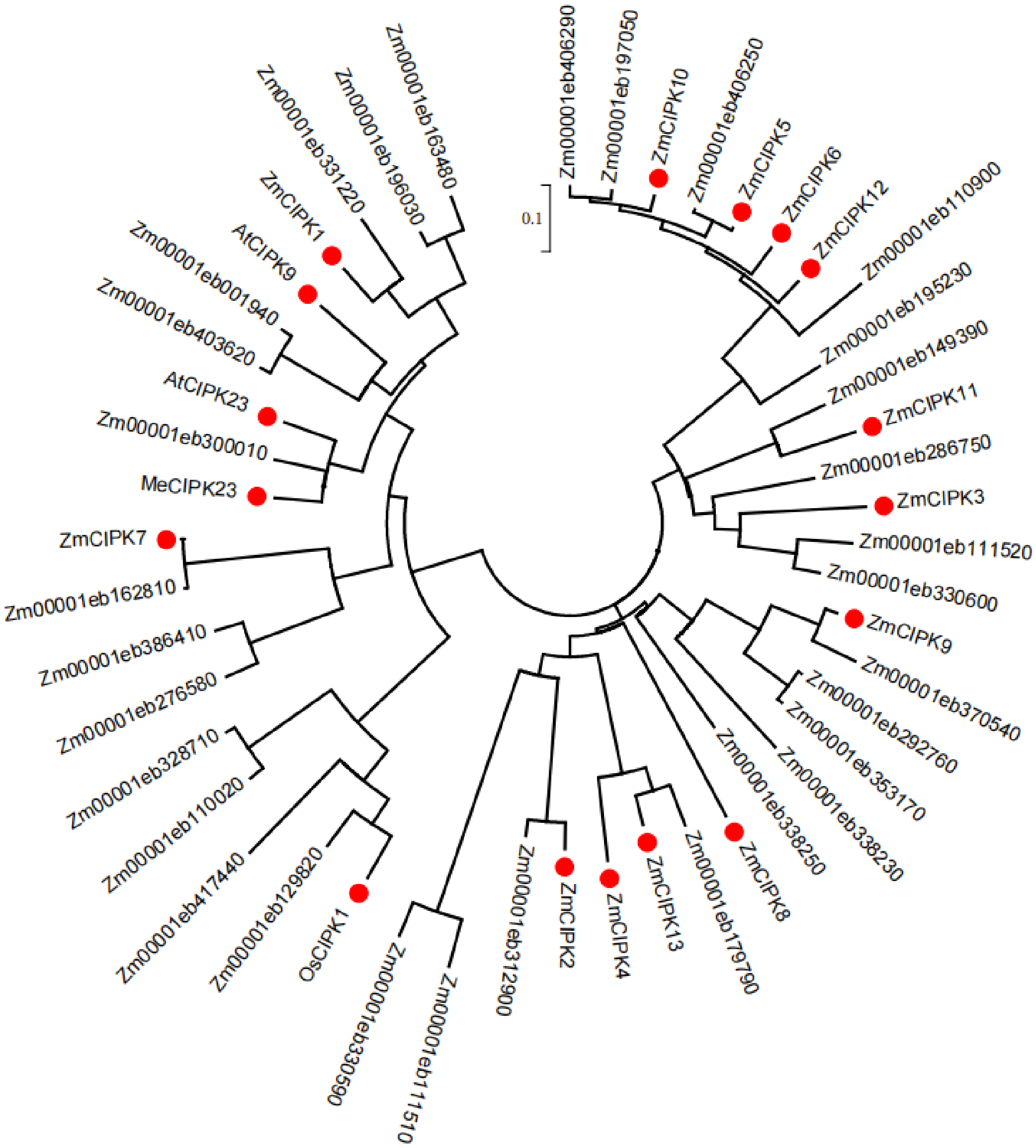

Fig. S10 Phylogenetic tree analysis of maize CIPK family genes and reported genes.

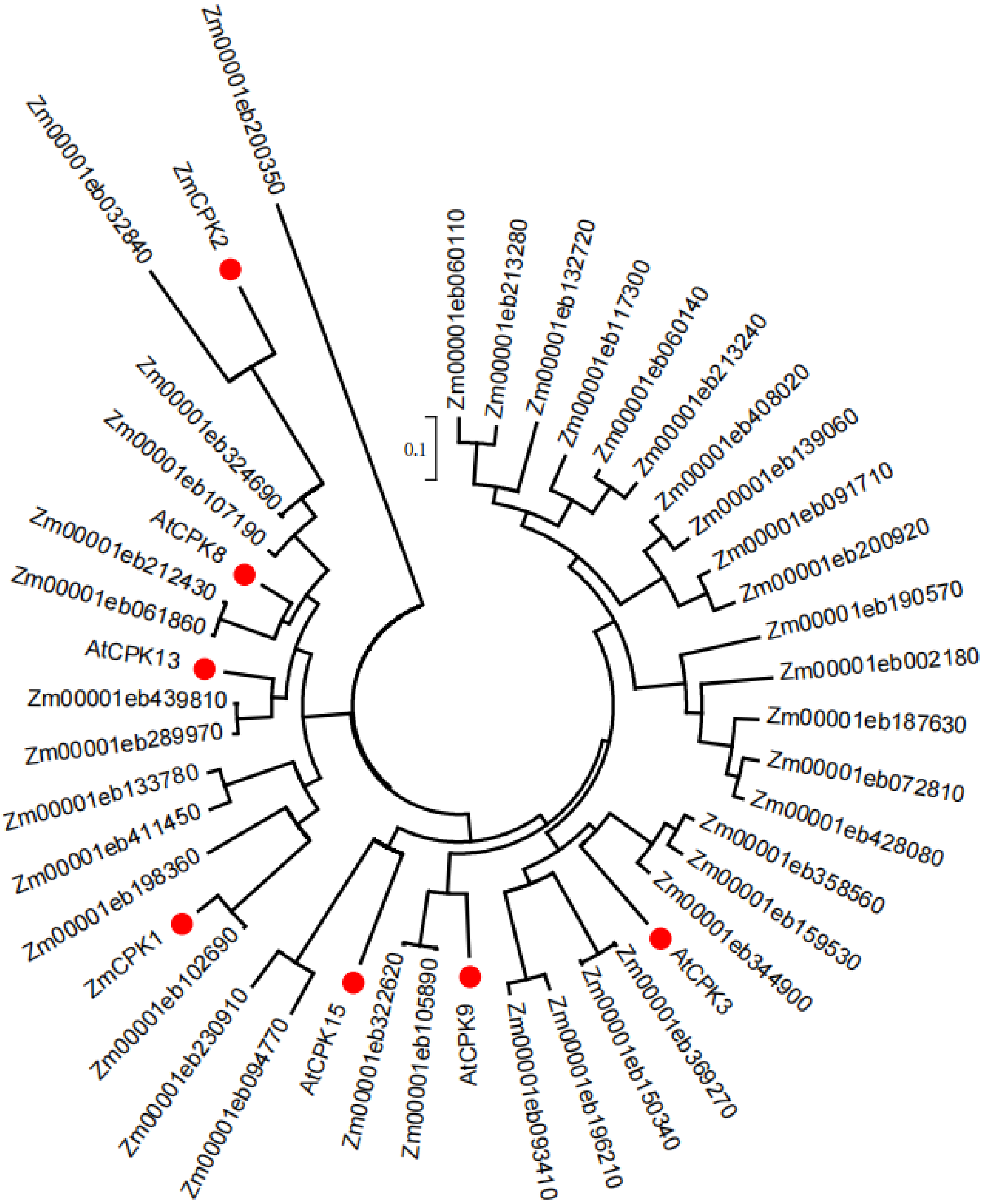

Fig. S11 Phylogenetic tree analysis of maize CPK family genes and reported genes.

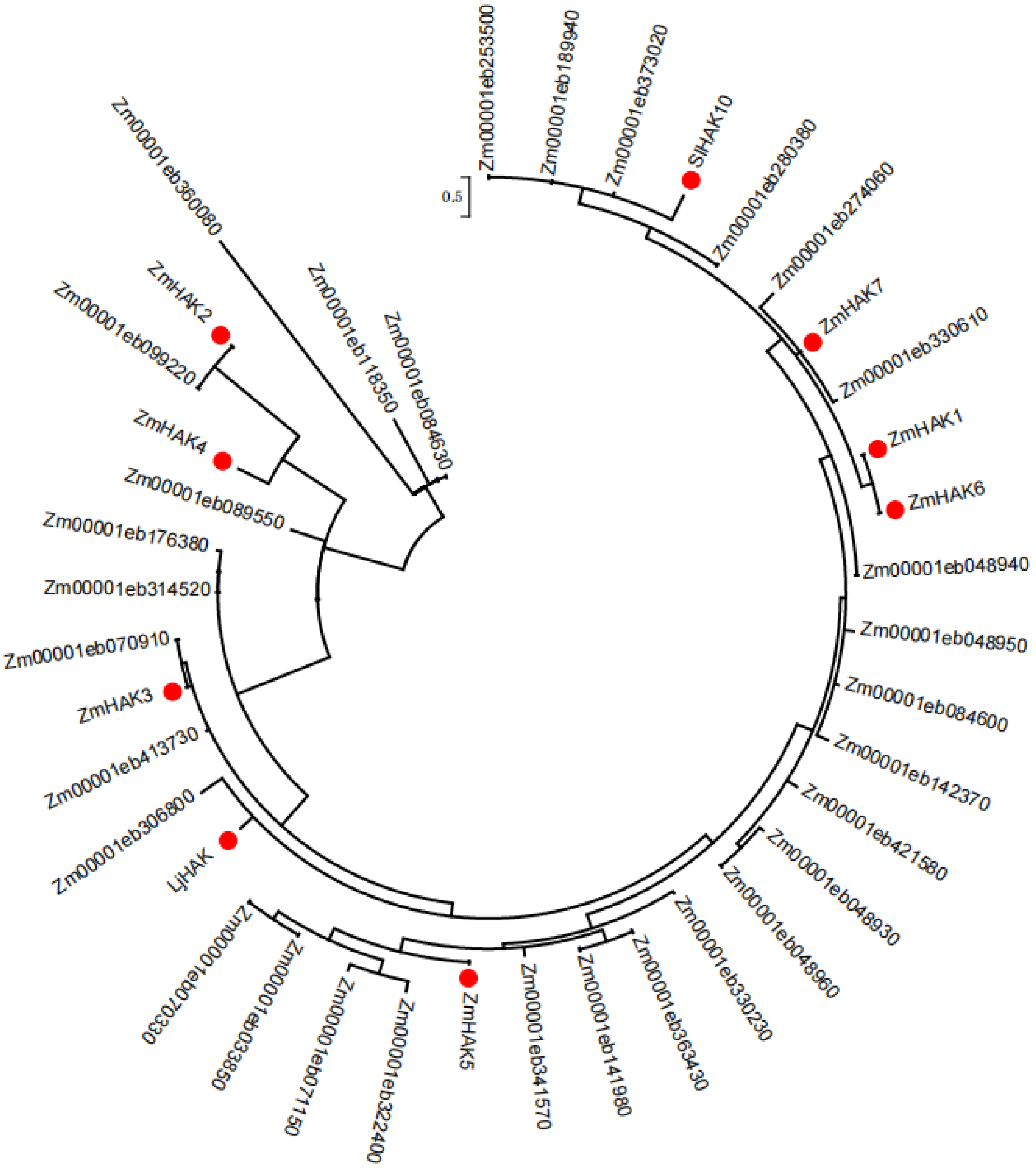

Fig. S12 Phylogenetic tree analysis of maize HAK/HKT/KUP family genes and reported genes.

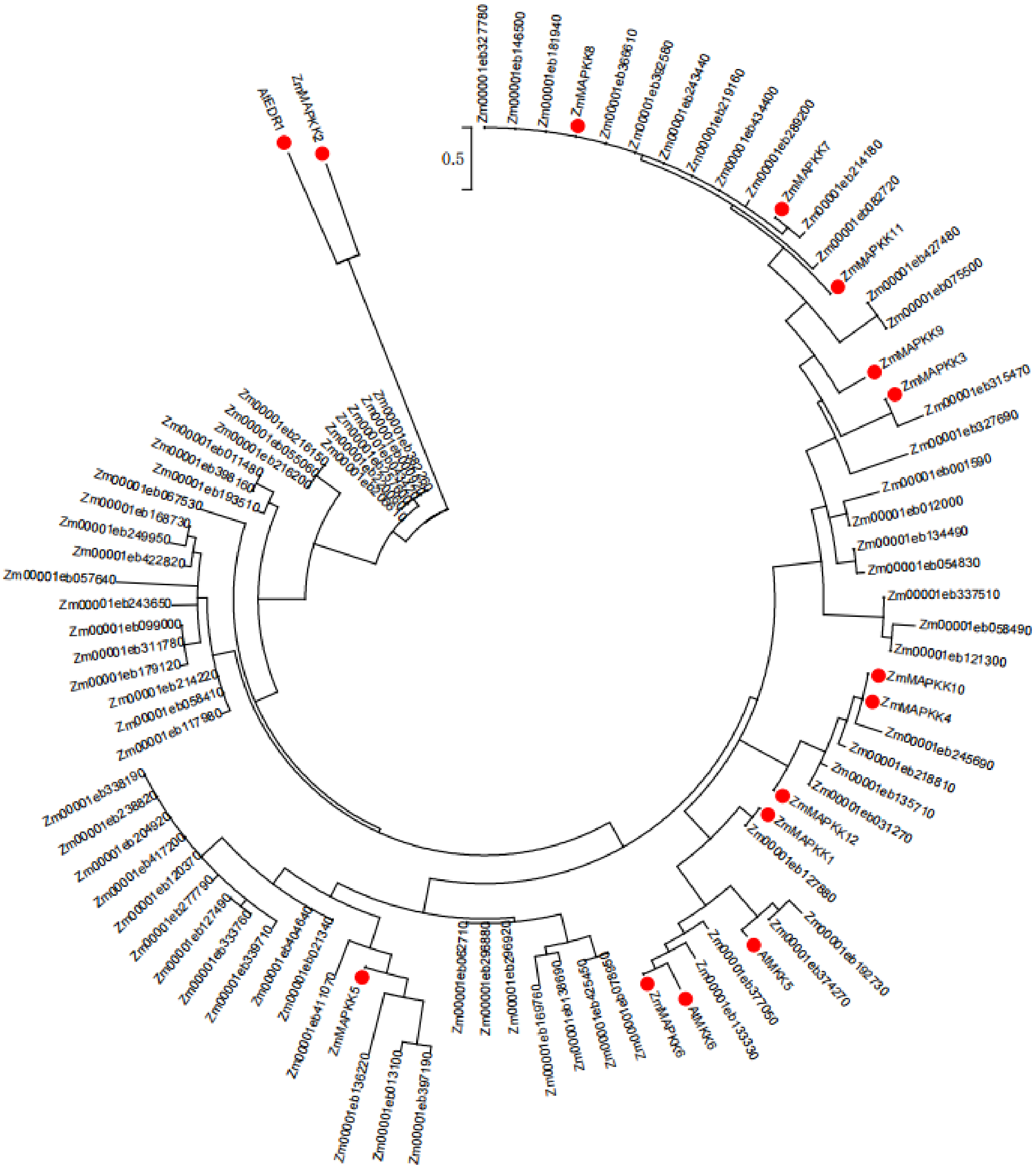

Fig. S13 Phylogenetic tree analysis of maize MAPKK family genes and reported genes.

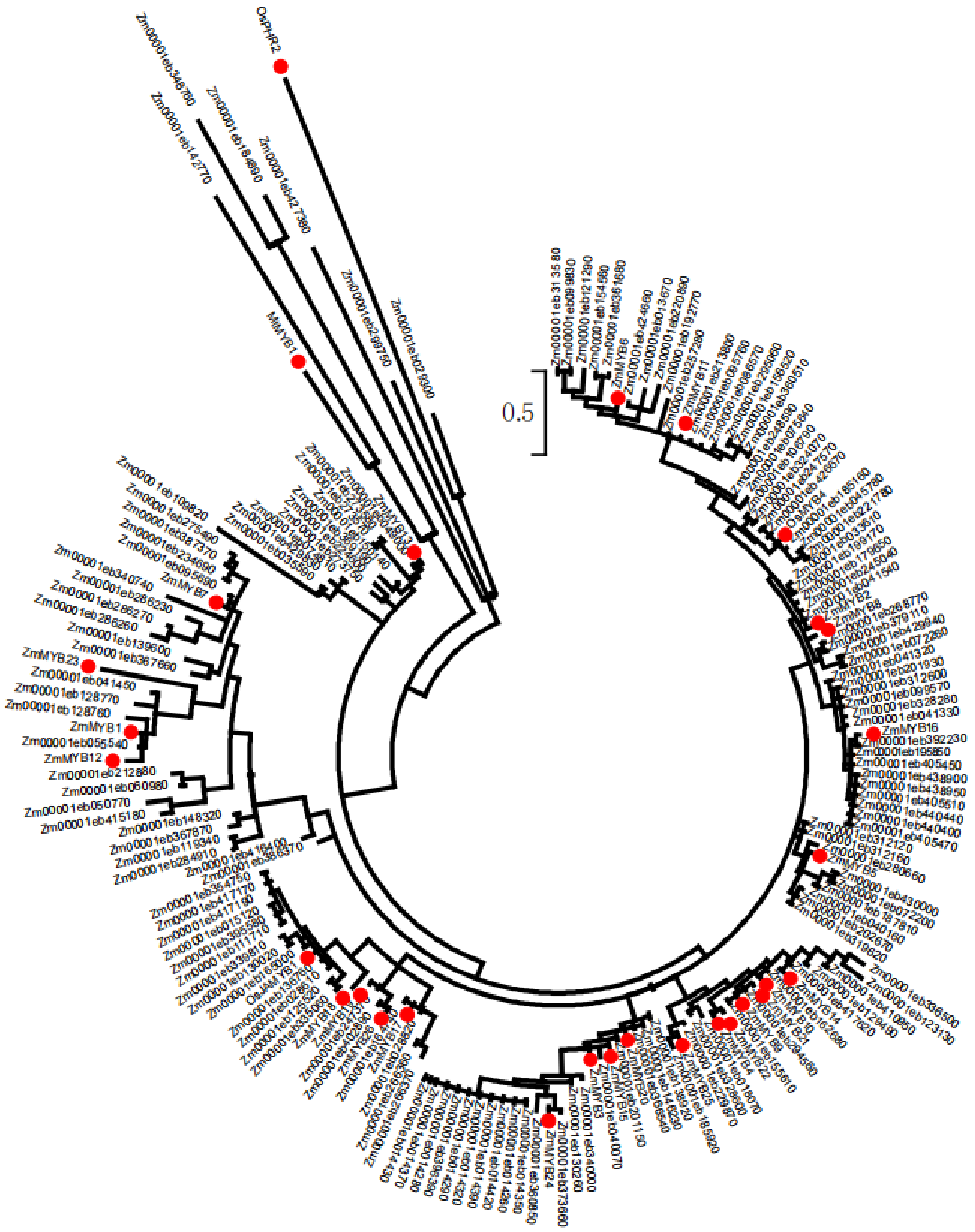

Fig. S14 Phylogenetic tree analysis of maize MYB family genes and reported genes.

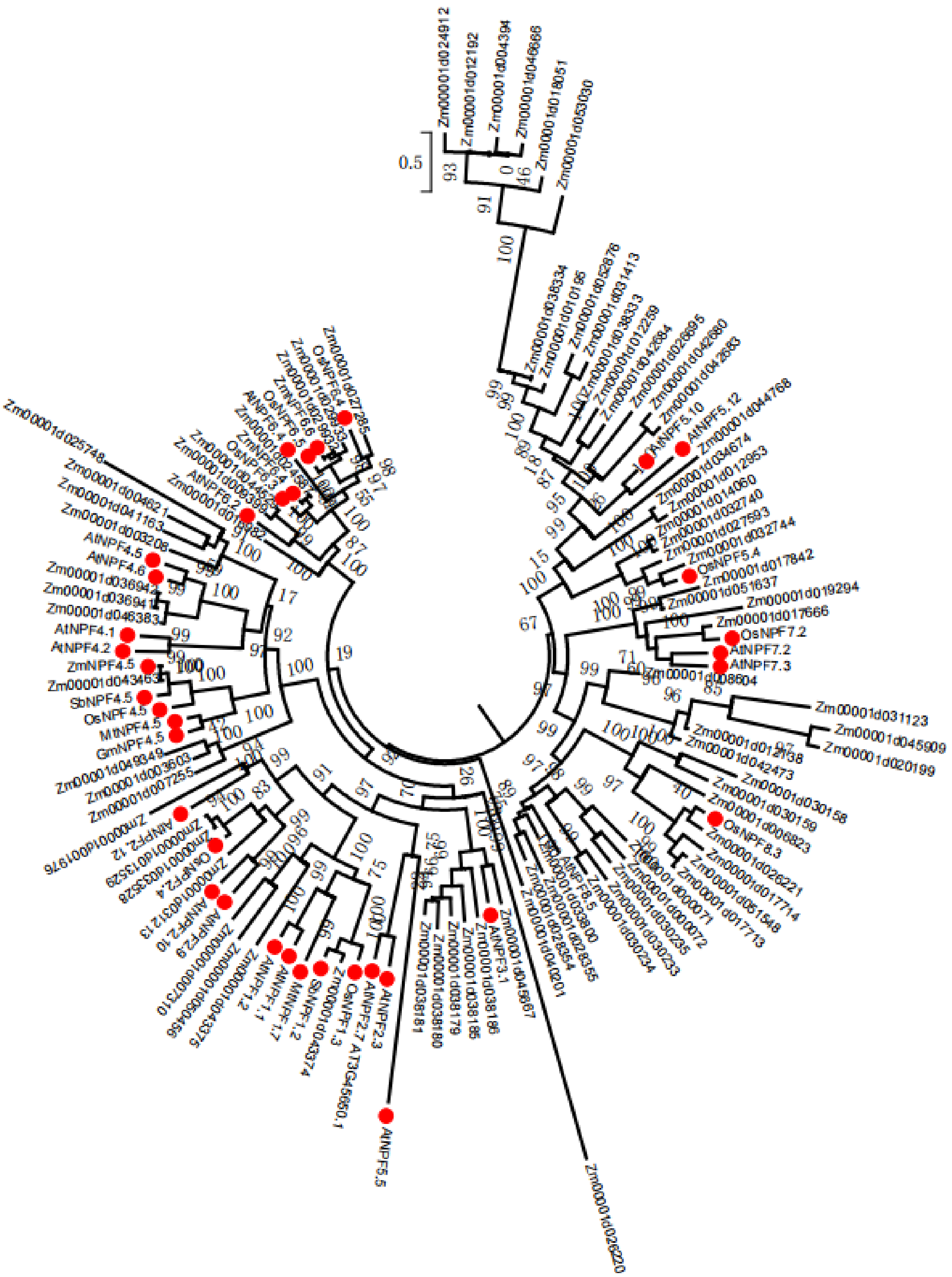

Fig. S15 Phylogenetic tree analysis of maize NPF family genes and reported genes.

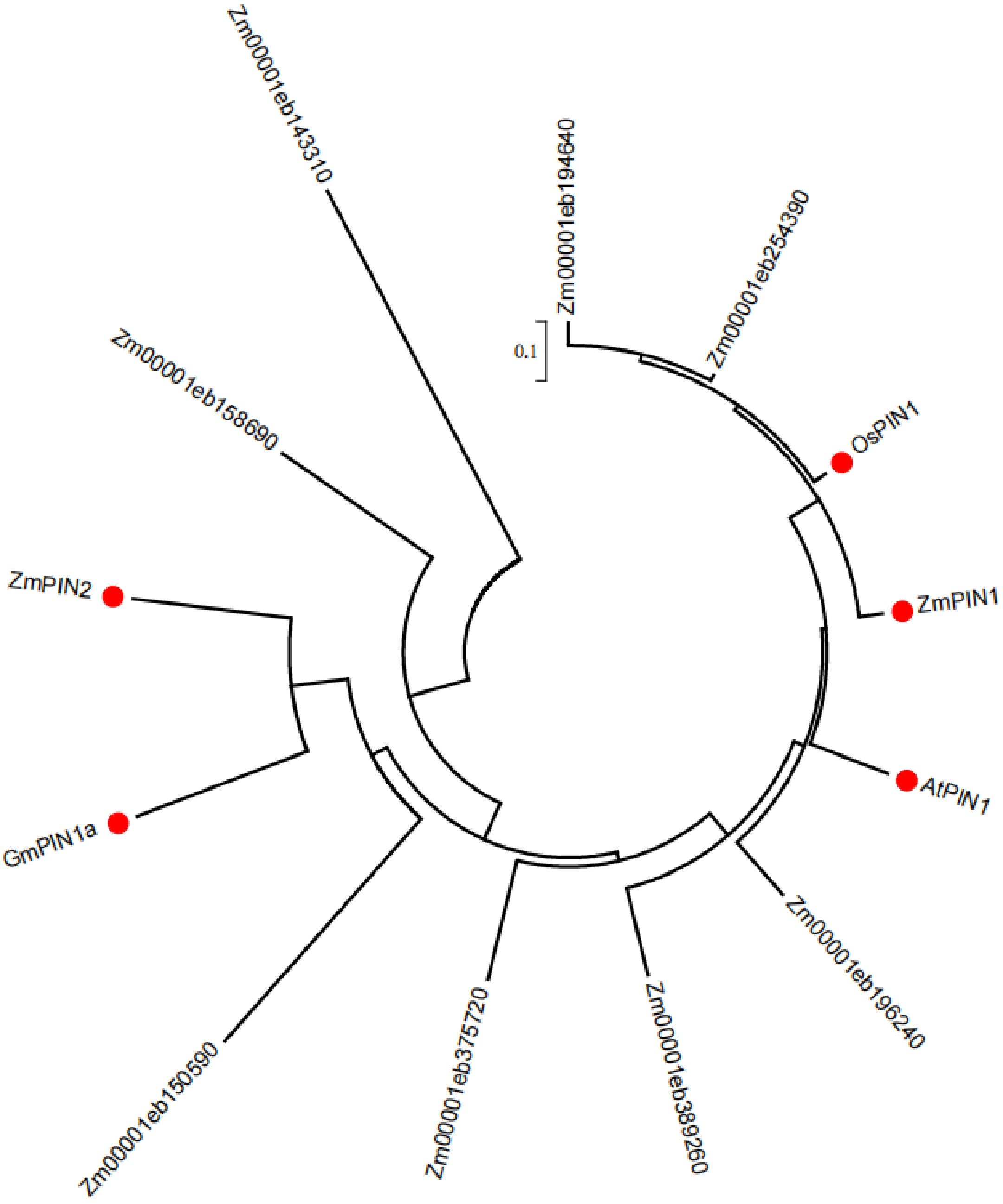

Fig. S16 Phylogenetic tree analysis of maize PIN family genes and reported genes.

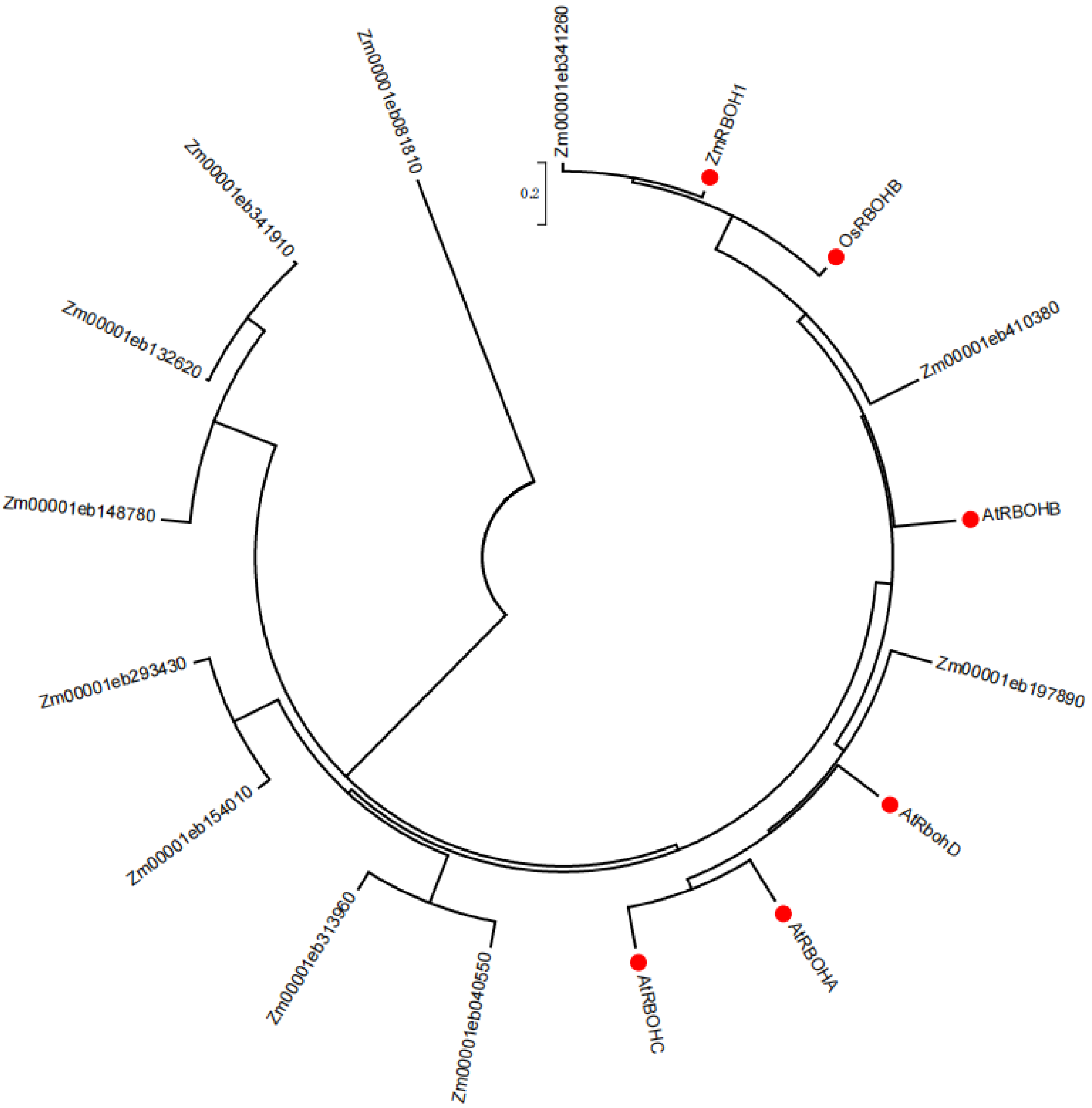

Fig. S17 Phylogenetic tree analysis of maize RBOH family genes and reported genes.

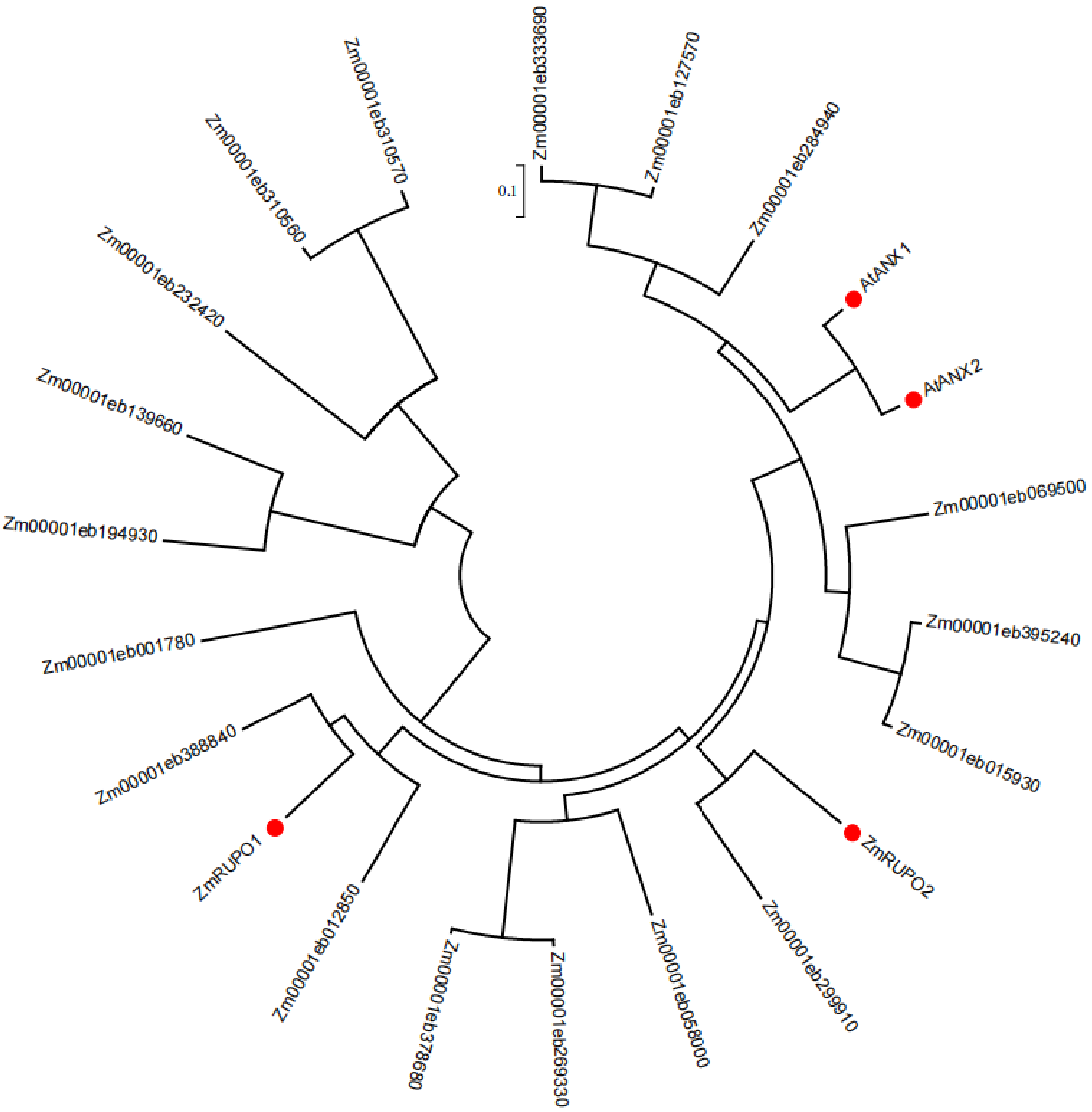

Fig. S18 Phylogenetic tree analysis of maize RUPO family genes and reported genes.
